# Evaluation of Moisture Damage Potential in Hot Mix Asphalt Using Polymeric Aggregate Treatment

**DOI:** 10.3390/ma15155437

**Published:** 2022-08-07

**Authors:** Arsalan Raza, Imran Khan, Rana Faisal Tufail, Jana Frankovska, Muhammad Umar Mushtaq, Abdellatif Salmi, Youssef Ahmed Awad, Muhammad Faisal Javed

**Affiliations:** 1 Department of Civil Engineering, Wah Engineering College, University of Wah, Wah Cantt 47040, Pakistan; 2 Institute of Environmental Sciences and Engineering, School of Civil and Environmental Engineering, National University of Sciences and Technology, Islamabad 44000, Pakistan; 3 Department of Civil Engineering, Graduate School of Engineering, Nagoya University, Nagoya 464-8603, Japan; 4 Department of Geotechnics, Faculty of Civil Engineering, Slovak University of Technology Bratislava, 811 07 Bratislava, Slovakia; 5 Department of Civil Engineering, COMSATS University Islamabad, Wah Campus, Mall Road Quaid Avenue, Wah Cantt 47040, Pakistan; 6 Department of Chemical Engineering, Wah Engineering College, University of Wah, Quaid Avenue, Wah Cantt 47040, Pakistan; 7 Department of Civil Engineering, College of Engineering, Prince Sattam bin Abdulaziz University, AlKharj 16273, Saudi Arabia; 8 Structural Engineering, Faculty of Engineering and Technology, Future University in Egypt, New Cairo 11835, Egypt; 9 Department of Civil Engineering, Abbottabad Campus, COMSATS University Islamabad, Abbottabad 22060, Pakistan

**Keywords:** moisture damage, hot mix asphalt, high-density polyethylene, SBR latex, indirect tensile strength test, indirect tensile modulus test, polymeric aggregate treatment

## Abstract

To enhance the moisture damage performance of hot mix asphalt (HMA), treating the aggregate surface with a suitable additive was a more convenient approach. In this research, two types of aggregate modifiers were used to study the effect of moisture damage on HMA. Three different aggregate sources were selected based on their abundance of use in HMA. To study the impact of these aggregate modifiers on moisture susceptibility of HMA, the indirect tensile strength test and indirect tensile modulus test were used as the performance tests. Moisture conditioning of specimens was carried out to simulate the effect of moisture on HMA. The prepared samples’ tensile strength ratio (*TSR*) and stiffness modulus (*Sm*) results indicated a decrease in the strength of the HMA after moisture conditioning. After treating the aggregate surface with additives, an improvement was seen in dry and wet strength and stiffness. Moreover, an increasing trend was observed for both additives. The correlation between TSR and strength loss reveals a strong correlation (R^2^ = 0.7219). Also, the two additives indicate increased wettability of asphalt binder over aggregate, thus improving the adhesion between aggregate and asphalt binder.

## 1. Introduction

Well-managed and proper road infrastructure is the strength of a country’s growth and prosperity. Pakistan is a developing country, and continuous efforts are being made to improve road quality. Poor drainage, heavy loading, and harsh weather conditions escalate road failures. Infiltration of moisture in pavement leads to loss of bonding between bitumen and aggregates in the hot mix asphalt (HMA) mixture; therefore, it is regarded as a major contributing factor in the degraded performance of pavement [1,2,3]. There are two types of moisture-related failure: (i) adhesive failure, which is the stripping of the asphalt film from the aggregate, and (ii) cohesion failure, which is loss of stiffness in a mixture [4,5,6,7,8]. The weakening of the adhesive bond, due to interaction with water leads to the phenomenon known as stripping, leading to reduced durability of HMA pavement [9]. Adhesive failure occurs at the binder–aggregate interface, while cohesive failure occurs within the asphalt [10,11]. Both mechanisms can be linked with the binder, the aggregate, or interactions between the former and the latter. Loss of strength in HMA occurs when the bond between binder and aggregate is weakened. Stripping begins at the bottom of the HMA layer and travels upward [12,13], leading to a gradual loss of strength over the years and the promotion of rutting and shoving in the wheel path [14]. Additionally, the adhesive bonding at the aggregate–binder interface is more likely to be affected by water than the cohesive failure in the binder [15]. Moreover, stripping is difficult to identify because surface indicators may require years to show [16]. To minimize moisture susceptibility in HMA, a barrier is provided on the aggregates in the form of coating, which can resist water while enhancing bonding with the asphalt binder. 

Stripping of asphalt pavements occurs at the molecular level. Stripping is thought to be associated with either one or both of the following two phenomena: (i) water can interact with asphalt binder to cause a reduction in cohesion which leads to reduced stiffness and strength of the HMA, and (ii) water can get between the asphalt film and the aggregate, break the adhesive bond, and strip the asphalt binder from the aggregate [17,18,19]. Several factors affect the adhesion of the asphalt binder to the aggregate, which includes interfacial tension between the asphalt binder and the aggregate, binder viscosity, chemical composition of the asphalt binder and aggregate, the surface texture of the aggregate, aggregate porosity, aggregate temperature, moisture content at the time of mixing aggregate, and aggregate cleanliness [20,21]. Moisture affects bituminous mixtures in several ways. The mix structure is weakened once moisture enters the mix [22]. The stiffness of the mix decreases and fails under cyclic traffic loading [23]. Six main mechanisms can cause the stripping of asphalt film from the aggregate surface that may act independently or collectively. These mechanisms include detachment, displacement, spontaneous emulsification, film rupture, pore pressure, and hydraulic scour [24,25,26].

Khurshid et al. [27] studied aggregate modification and concluded that coating aggregate with HDPE increases aggregate impact and abrasion resistance, improving the coated aggregate’s wear resistance and strength properties. Using a modified mixture of 8% shredded HDPE improves the Marshall stability of the mixture by 70%, improving the load-carrying capacity and rutting resistance. Zhang et al. [28] investigated the influence of three different polyethylene wax additives, i.e., high density, low density, and oxidized polyethylene wax, in asphalt mixtures. These additives were selected according to their molecular weight (*M_w_*), and results indicated that the moisture resistance capacity of asphalt mixtures was improved with an increase in *M_w_* and enhanced mixture stability at a lower temperature. Additionally, oxidized polyethylene wax was more suitable for having a uniform microstructure. Gibreil and Feng [29] researched the effects of HDPE and crumb rubber powder (CR_p_) on the properties of HMA. Adding CR_p_ and HDPE improved the physical and Marshall properties of HMA mixtures with a significant increase in resistance to moisture damage and permanent deformation. Al-Hadidy and Yi-qiu [30] also indicated that adding polyethylene modifiers to asphalt mixtures could improve resistance to deformation and moisture-induced damage at moderate and elevated temperatures. 

Mishra and Singh [31] determined that binder–aggregate bonding increases due to the increased contact area between polymer-modified aggregate and binder. Coating polymer also helps reduce voids, preventing binder oxidation and moisture absorption due to entrapped air. In another study, Wasiuddin et al. [32] evaluated the moisture sensitivity of two types of HMA mixtures made with two types of aggregate with and without styrene–butadiene–rubber (SBR) treatment for moisture-induced damage potential. Coating the SBR improved the aggregate surface, increased the wettability of asphalt binder over the aggregate, and changed the aggregate surface from hydrophilic to hydrophobic. Kim et al. [33] presented an approach to help understand moisture damage mechanisms and evaluate the effects of moisture-damage resisting agents.

To this end, various cases of performance testing of HMA samples induced by moisture damage and several fundamental property measurements (stiffness, strength, toughness, and bonding energy) of mixture components were conducted. Testing data and analyses demonstrated that anti-stripping additives contributed to moisture damage resistance due to the synergistic effects of mastic stiffening, toughening, and advanced bonding characteristics at mastic-aggregate interfaces [34]. Nejad et al. [35] studied the effects of nanomaterial coating, namely Zycosoil, on the moisture damage of asphalt mixtures. Their study showed that aggregate coating with a suitable agent caused an increase in the ratio of wet/dry values of indirect tensile strength and indirect tensile fatigue in treated samples compared to the control mix. Nejad et al. [36] also established that aggregate coating with a suitable agent is an appropriate method that obtains a resistant mixture of asphalt binder and aggregate against moisture damage. Polyethylene coating altered the aggregate surface from hydrophilic to hydrophobic, thereby increasing the wettability of the asphalt binder over the aggregate. Kanitpong and Bahia [37] suggested that designing a binder with enhanced adhesive and cohesive properties could strengthen the HMA mixture against moisture damage.

Chen and Huang [38] evaluated the moisture vulnerability of HMA through Superpave indirect tensile test and simple performance tests. In this study, Superpave indirect tensile test (IDT) and simple performance test (SPT) was undertaken to assess the effects of moisture damage on an HMA mixture using coarse gravels to make mixtures with equivalent aggregate gradations. HMA specimens were conditioned using the freeze-thaw (*F-T*) cycle and 500 and 1000 cycles of pore pressure pulses with Moisture Induced Stress Tester (*MIST*). This study proved that moisture damage in HMA was increased with subsequent increases in *F-T* and *MIST* cycles. Albayati & Mohammed [39] investigated the influence on the mechanical properties of HMA by adding SBR polymer. Experimental results showed that SBR polymer-modified mixture improved behavior against fatigue and permanent deformation with improved elastic properties. 

Furthermore, it is evident from the past studies that many researchers have employed local field conditions to impart modification in HMA mixtures by using different additives. In a country such as Pakistan, the problems relating to moisture damage are more severe, mainly due to poor quality control. Therefore, the current study involves the modification of HMA mixtures by incorporating HDPE and SBR latex with various locally available materials. A thorough comparison between modified HMA using HDPE, SBR latex, and conventional mixture is drawn. For this purpose, three main aggregate types are used in this study, primarily used in pavement construction in Pakistan. This research could be useful for the practitioners and researchers dealing with the latest materials evaluation and screening protocols/techniques to form moisture-resistant HMA mixtures.

## 2. Methodology

**Figure 1:**
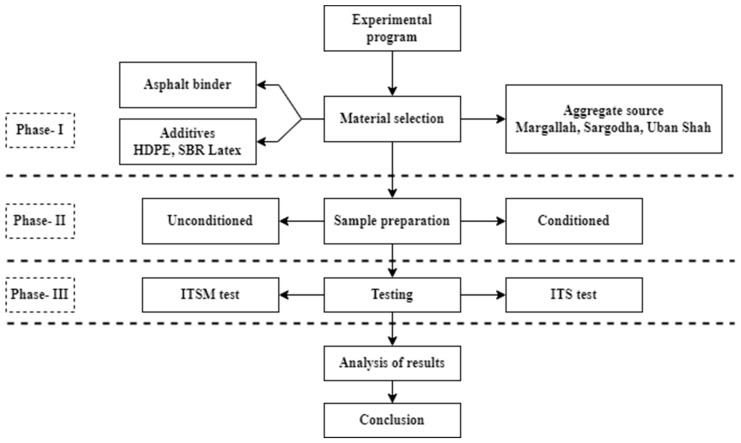
Flowchart of the experimental design.

A three-phase experimental program was designed to achieve this study’s objectives. Phase-I determined the physical properties of selected materials, while Phase-II involved preparing and conditioning conventional and modified HMA mixtures with subjected additives. Phase-III included determining moisture susceptibility and the mechanical properties of HMA mixtures using the Indirect Tensile Strength Test and Indirect Tensile Modulus Test (Figure 1).

### 2.1. Materials

In Phase-I, various materials are selected; for instance, 60/70 (PG 64-22) penetration-grade asphalt binder was obtained from a local refinery (Attock refinery) and tested in the laboratory. Conventional and Superpave rheological experiments were performed to ascertain the viscoelastic and physical properties of the binder. Conventional test results involved penetration at 25 °C (100 g, 5 s, and 0.1 mm), ductility at 25 °C, specific gravity (60/70 grade asphalt binder), and softening point, which were recorded as 62 dmm, 101 cm, 1.02, and 51 °C, respectively. Moreover, a master curve based on field temperature and loading patterns from a frequency sweep test was used to predict the viscoelastic behavior of the asphalt binder. The optimum binder content was found only using control mixes of all three aggregate sources. The Marshal mix design method was used to calculate the optimum binder content of Margallah (A_M_), Sargodha (A_S_), and Uban Shah (A_U_) was found to be 4.17%, 4.05%, and 4.09%, respectively. 

**Figure 2:**
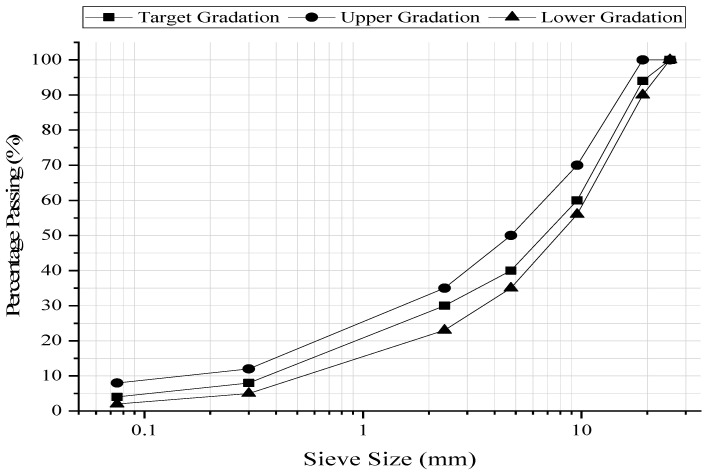
Gradation curve.

The general practice in preparing HMA mixtures is using asphalt binder and aggregates procured from different quarries. The selection of a particular quarry depends upon the distance from the intended construction site. Therefore, HMA mixtures were prepared using one particular asphalt binder and different types of limestone aggregates. For this purpose, three (A_M_, A_S,_ and A_U_) frequently used types of aggregates in Pakistan were employed to fabricate HMA mixtures. The physical properties of the aggregates are provided in Table 1. It is important to note that NHA Class-A wearing course gradation was adopted for this research because it is a coarser gradation and is suspected to be more prone to moisture damage than NHA Class-B wearing course gradation (Figure 2). Moreover, chemical compositions of aggregates were also determined and are presented in Table 2. 

**Figure 3:**
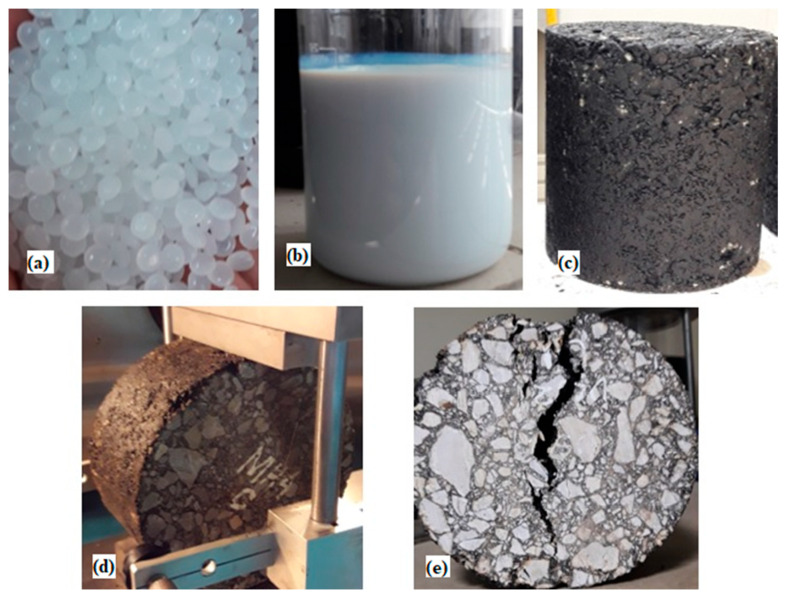
Materials and testing: (**a**) HDPE pellets; (**b**)SBR Latex; (**c**) prepared sample; (**d**) ITS test; and (**e**) specimen after test.

In this study, SBR latex and HDPE are employed as additives (Figure 3a,b). SBR latex is a carboxylated styrene–butadiene copolymer latex used with other binding materials to improve a mixture’s bond strength. The appearance of the SBR latex used in this study was a whitish viscous emulsion. The Brookfield viscosity, pH, and particle size were <300 cps, 8.5–9.5, and 175 nm, respectively. Furthermore, HDPE, a polyethylene thermoplastic produced from petroleum, was utilized as an additive. The mechanical characteristics of HDPE were evaluated; the density, tensile strength, flexural modulus, and elongation percentage at break were 0.955 g/cm^3^, 27.1 MPa, 1371 MPa 563, respectively. 

### 2.2. Testing Procedures

In the case of HDPE, the aggregates were heated to 190 °C to achieve a suitable temperature for melting and mixing, and HDPE was added at 0.3%, 0.4%, and 0.5% by aggregate weight to form a thin film over the aggregate. For SBR latex, aggregates were pre-heated to a temperature of 130 °C, and SBR latex was added at 1%, 2%, and 3% by aggregate weight and was thoroughly mixed with aggregate. 

In this research, the samples were prepared using a gyratory compactor (Figure 3c). Bitumen was added to the modified aggregate at 160 °C and mixed until it was uniform in the mixture. The mixture was then placed in the mold of a gyratory compactor, with 150-mm diameter and 200-mm height. The mold in the compactor was gyrated at an angle of 1.25°, and sample compaction was achieved with 600 kPa pressure. A good amount of loose asphalt mixture was added to the mold. The mold was then placed in the gyratory compactor, and the height of the sample was closely monitored. The compactor terminated the gyration after the required height was achieved. Air voids were kept up to 7% in the mixture using the gyrate-to-height feature.

The moisture conditioning of the samples was carried out as per AASHTO T283-03 [40]. The samples were divided into two subsets of three: one subset was tested without conditioning, and one subset was tested after moisture conditioning. Unconditioned samples were stored at room temperature (25 °C) for 24 h before testing. The sample was placed in the vacuum container for conditioned samples, and a vacuum pressure (13–67 kPa) was applied for 5 to 10 min. The degree of saturation (*S*′) was determined by comparing the volume of air voids (*V_a_*) and volume of water absorbed (*J*′) using the following equation:

(1) $$S^{\prime }=\frac{J^{\prime }}{V_{a}}\times 100$$

*S*′ was found to be between 70 and 80%. The samples were then placed in the freezer at −18 °C for a minimum of 16 h. The samples were then removed from the freezer and placed into a water bath at 60 °C for 24 h. The samples were then removed from the water bath, kept at 25 °C for 2 h, and tested.

A test to determine the resistance of compacted hot mix asphalt to moisture-induced damage was conducted per AASHTO T283-03. After removing the samples from the water bath, they were placed between the bearing plates of the universal testing machine (Figure 3d). Loading strips of steel were placed between the sample and bearing plates. A constant 50-mm/min load was applied until the sample was cracked (Figure 3e). The maximum load (*P*) was recorded, and the machine was stopped.

(2) $$S=\frac{2000P}{\pi \times tD}$$

where *S* is the tensile strength, *P* is the maximum load applied, t is the mean thickness of the specimen, and *D* is the diameter of the specimen.

The tensile strength ratio (*TSR*) was determined using the following equation:

(3) $$TSR=\frac{S_{conditioned}}{S_{unconditioned}}\times 100$$

The indirect tensile modulus test provides a stress–strain relationship of the asphalt mixes under dynamic loading. All samples with diameter of 150 mm and thickness of 75 mm were tested for stiffness modulus at 25 °C temperature. The testing was carried out by applying 20% of square wave loading of indirect tensile strength test (i.e., loading = 0.1 s and rest = 0.9 s) on the diametrical plane of the specimen to obtain stiffness moduli. The detailed parameters can be found in prior research [41,42]. The stiffness modulus (*S_m_*) is calculated using the following equation.

*Sm = F ×* (*μ +* 0.27)/(*h × Z*) (4)

where *F* is maximum loading, *μ* is the Poisson ratio, *h* is specimen height, and *Z* is horizontal deformation.

## 3. Results and Discussion

### 3.1. Indirect Tensile Strength 

**Figure 4:**
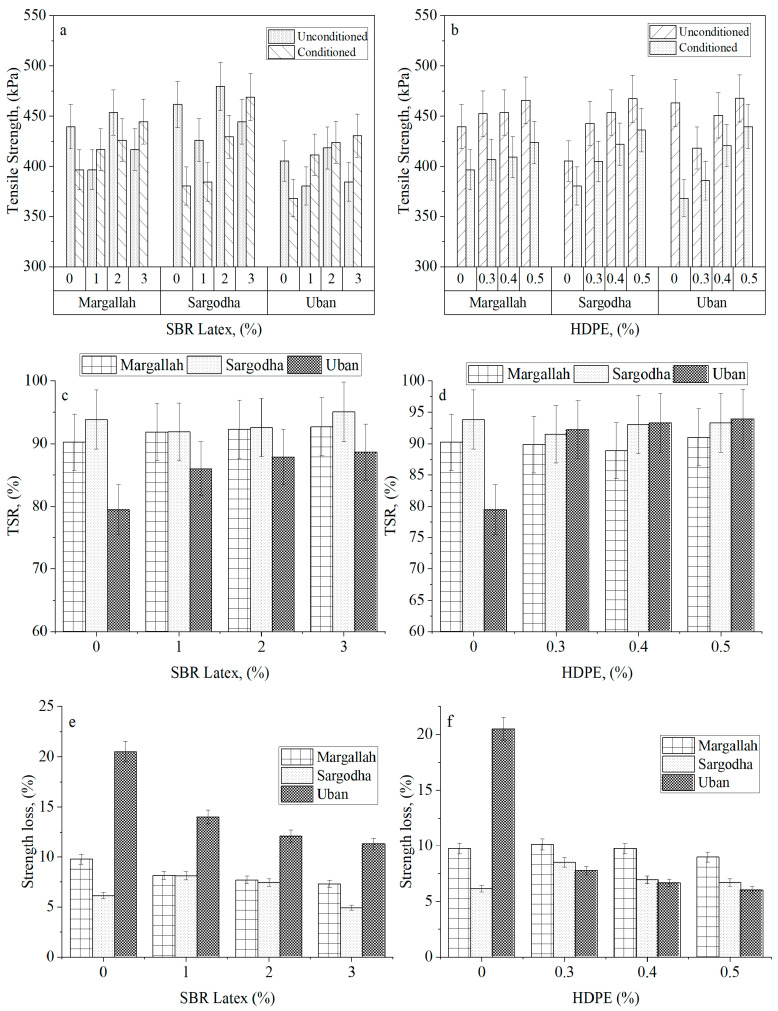
Results of (**a**) ITS test modified with SBR Latex, (**b**) ITS test modified with HDPE, (**c**) TSR of SBR Latex modified samples, (**d**) TSR of HDPE modified samples, (**e**) strength loss in SBR Latex modified samples, and (**f**) strength loss in HDPE modified samples.

An increasing trend in tensile strength of all aggregate sources was observed by increasing the percentage of both polymers in the mixture. A possible explanation for this phenomenon can be explained in terms of surface free energy. As explained by Wang et al. [43], adding polymer to the aggregate mix tends to decrease the total surface free energy of the aggregate, hence improving the wettability of the binder over the coated aggregate. Yalghouzaghaj et al. [44] proved that increasing the percentage of polymers reduces the surface free energy to the same level. The same trend can be observed in Figure 4, such that, as the percentage of HDPE and SBR latex increases, the tensile strength values for all the aggregate sources become nearly equal.

The influence of mineralogy of different aggregates can also be inferred from the given results (Figure 4). For instance, Sargodha aggregate contains 4% silica content, which is the highest among all three sources (Table 2). It was observed that a higher percentage of silica content causes a reduction in tensile strength of the mix since silica imparts a reduction in bond strength of the asphaltic mixture [45,46,47,48]. Adding SBR latex and HDPE altered the A_S_ surface from hydrophilic to hydrophobic. I.e., the conditioned tensile strength of A_S_, having 4% silica content, was observed to be 6% higher than the A_U_, having no silica content, thus showing the influence of silica content on the mechanical characteristics of HMA. Further increasing the percentage of SBR latex to 2% and 3% tends to increase the Sargodha aggregate’s tensile strength. 

The effect of moisture damage on the tensile strength of HMA is presented in Figure 4. It is evident from the results that the ITS values of dry samples are higher than conditioned samples. It was expected because water reduces the bonding between aggregate and binder under loading. Mixtures treated with SBR latex have higher tensile strength in conditioned and unconditioned samples than HDPE in unconditioned and conditioned states. This improved performance could indicate that SBR latex provides a better surface for binder wettability, which was also validated by the investigation of Wasiuddin et al. [32].

Treating the aggregate tends to increase its performance in dry and wet states. A possible explanation for this phenomenon could be the reduction in moisture permeability of the mixture. Although, to some researchers, treating the aggregate with the polymer may seem unnecessary with regards to TSR values, it may be pointed out that the overall strength gain increased at an average of 14% and 17%, just by adding up to 0.5% HDPE and 3% SBR latex, respectively. This observation advocates for the use of polymer in HMA mixtures.

From Figure 4, in the case of A_U_ aggregate, the TSR value of the control mix was closer to the minimum criteria provided by Superpave, i.e., 80%. Adding SBR latex and HDPE significantly improved the TSR of A_U_ by 11.55% and 18%, respectively, compared to their control mix, indicating an enhanced moisture damage resistance of the mixture.

### 3.2. Indirect Tensile Modulus Test

The stiffness modulus (*M_R_*) can be explained as the estimate of the stress–strain relationship of asphalt pavement. Increased strain can be experienced with a decrease in M_R_ after moisture conditioning due to traffic loading, resulting in an increased tendency towards rutting and fatigue cracking. 

**Figure 5:**
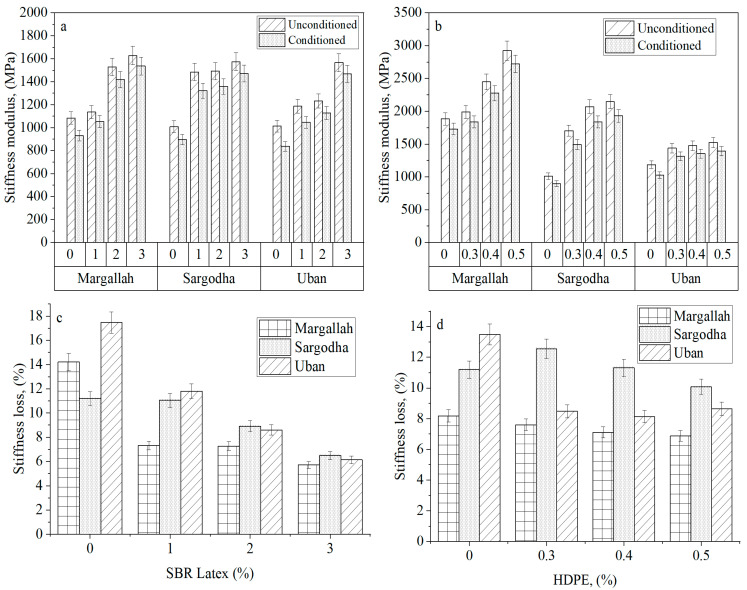
Results of (**a**) ITMT modified with SBR Latex, (**b**) ITMT modified with HDPE, (**c**) stiffness loss in SBR Latex modified samples, and (**d**) stiffness loss in HDPE modified samples.

After moisture conditioning, mixtures treated with SBR latex and HDPE have shown stiffness decay. Figure 5 indicates significant improvement in *S_m_* with an increase in the percentage of SBR latex and HDPE in conditioned samples. This increase in *S_m_* shows that HMA with control mix is more susceptible to cracking and permanent deformation than modified aggregates. Additionally, the higher conditioned stiffness modulus of SBR-latex and HDPE mixtures could be correlated to improved interlocking between the mixture particles. Interlocking strength has been improved because of the penetration of SBR latex and melted HDPE between the spaces in the aggregate particles. This formation of interfacial film results in a continuous connection between aggregates, improving the overall interlocking strength of the mixture [49].

Figure 5 shows significant improvement in *S_m_* with the increase in the percentage of both SBR latex and HDPE in conditioned samples, indicating that incorporating HDPE into the aggregate tends to improve the overall flexibility of the mixture more than compared to SBR latex, which was also endorsed by Bargegol et al. [50]. The HDPE sample has a higher stiffness modulus than samples containing SBR latex. A possible justification could be that HDPE possesses higher tensile strength, and the inclusion of HDPE in the sample increases the overall flexibility of the mixture. A_U_’s stiffness modulus values are lower than A_M_ and A_S_, indicating that A_U_ is more susceptible to moisture damage without adding modifiers, i.e., 0%. Another interesting factor highlighted is the percentage loss in stiffness of conditioned and unconditioned mixtures. It has been observed that the addition of SBR latex and HDPE had an inverse relation with percentage loss in stiffness, i.e., increasing the amount of polymer in the mixture tends to decrease its overall stiffness loss.

### 3.3. Correlation between ITS and ITMT

**Figure 6:**
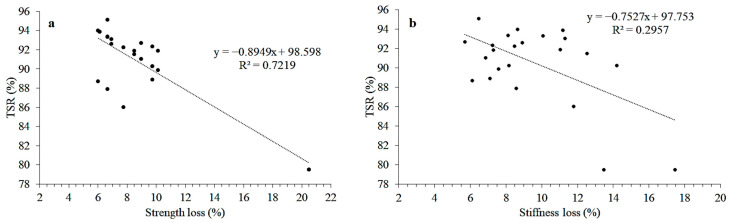
Relation between (**a**) TSR and Strength loss and (**b**) TSR and Stiffness loss.

Resistance to moisture damage is indicated in terms of TSR values, whereas the wet-to-dry ratio in ITMT demonstrates a loss of stiffness. The relationship between TSR, stiffness loss, and strength loss was checked. Figure 6 expresses two relationships: between TSR and strength loss, and TSR and stiffness loss. It was observed that strength loss and TSR exhibit a strong correlation (R^2^ = 0.7219), further indicating that an increase in strength loss can reduce the TSR of the HMA. Although the devised correlation was weak (R^2^ = 0.2957) between TSR and stiffness loss, an inverse relation was observed from the trendline. A logical trend indicates an increase in stiffness loss values with a decrease in TSR values, pointing to increased moisture susceptibility of the mixtures. This weak correlation (R^2^ = 0.2957) may be categorized due to the fact that the number of data points and other parameters, such as the number of additives, percentage of additives, number of aggregate sources, and test temperatures, are fewer [51].

A single-factor ANOVA test (α = 0.05) was conducted to validate the statistical significance of the variation in the values within the mixtures and between them. The results of the ANOVA tests are provided in Table 3. Based on the analysis, the data for both tests were statistically significant (*F_crit_* < F; *p*-value < 0.05). So, it is safe to say that ITS and ITMT are good indicators to point towards the moisture damage potential of a mixture.

## 4. Conclusions

This research attempted to study the behavior of treated aggregates with conventional asphalt binder. The present research focused on methods to minimize the moisture damage potential of HMA. For this purpose, the aggregate surface was coated with suitable additives to obtain a moisture-resistant mixture. Based on the results, the following conclusions are obtained:

- All conditioned samples have lower tensile strength values as compared to unconditioned samples. An increase in the percentage of HDPE and SBR latex also increased the strength of treated aggregate samples compared to their control-mix samples. Increasing the percentage of both additives caused the TSR values to improve significantly for all three aggregate sources, especially in the case of A_U_, showing improved moisture sensitivity.
- Moisture conditioning of samples reduced their stiffness value considerably compared to their respective unconditioned samples. Stiffness moduli increased for all three aggregate sources after aggregate coating. An increase in stiffness indicated that additives changed aggregate behavior from hydrophilic to hydrophobic.
- The comparison revealed a strong correlation (R^2^ = 0.7219) between TSR and strength loss and a weak correlation between ITS and stiffness loss (R^2^ = 0.2957). However, a logical trend was observed, indicating increased strength and stiffness loss as the TSR decreased.

This study shows that aggregate modification could improve HMA properties. The economic aspect needs to be studied for a full-scale industrial process. Also, the present research work may be carried out with a change in testing temperatures to study the behavior at different temperatures.

## Figures and Tables

**Table 1:** Properties of aggregate.

Source	ID	Sp. Gravity (G_sb_)	Water Absorption (%)	Elongation (%)	Flakiness (%)	Los-Angeles Abrasion Value	Aggregate Crushing Value	Aggregate Impact Value
Margallah	A_M_	2.637	0.78	3.3	6	21	27	32
Sargodha	A_S_	2.642	0.50	7	5.5	22.5	25	30
Uban Shah	A_U_	2.640	0.59	15	12	22	29	33

**Table 2:** Chemical composition of aggregates.

Component	Chemical Formula	Aggregate Source (%)		
		A_M_	A_S_	A_U_
Carbonate & Calcite	CaCO_3_ and CaO	96	94	98
Hematite	Fe_2_O_3_	0.5	-	2
Quartz	SiO_2_	1.5	3.9	-
Clay	-	2	-	-

**Table 3:** Single- Factor ANOVA test.

Source of Variation	SS	df	MS	F	*p*-Value	*F_crit_*
(i) For ITS						
Between Mixes	23,282.44	3	7760.812	12.69663	4.13 × 10^−6^	2.81647
Within Mixes	26,894.98	44	611.2496			
Total	50,177.42	47				
(ii) For ITMT						
Between Mixes	2,872,054	3	957,351.2	5.61978	0.002376	2.81647
Within Mixes	7,495,565	44	170,353.7			
Total	10,367,618	47				

## Data Availability

All data, models, and code generated or used during the study appear in the submitted article.
